# Mitral Valve Stenosis after Open Repair Surgery for Non-rheumatic Mitral Valve Regurgitation: A Review

**DOI:** 10.3389/fcvm.2016.00008

**Published:** 2016-04-21

**Authors:** Muhammad Shabsigh, Cassidy Lawrence, Byron R. Rosero-Britton, Nicolas Kumar, Satoshi Kimura, Michael Andrew Durda, Michael Essandoh

**Affiliations:** ^1^Cardiothoracic Anesthesiology Division, Department of Anesthesiology, The Ohio State University Wexner Medical Center, Columbus, OH, USA

**Keywords:** functional mitral stenosis, iatrogenic mitral stenosis, mitral valve repair surgery, intraoperative echocardiography, annuloplasty

## Abstract

Mitral stenosis (MS) after mitral valve (MV) repair is a slowly progressive condition, usually detected many years after the index MV surgery. It is defined as a mean transmitral pressure gradient (TMPG) >5 mmHg or a mitral valve area (MVA) <1.5 cm^2^. Pannus formation around the mitral annulus or extending to the mitral leaflets is suggested as the main mechanism for developing delayed MS after MV repair. On the other hand, early stenosis is thought to be a direct result of an undersized annuloplasty ring. Furthermore, in MS following ischemic mitral regurgitation (MR) repair, subvalvular tethering is the hypothesized pathophysiology. MS after MV repair has an incidence of 9–54%. Several factors have been associated with a higher risk for developing MS after MV repair, including the use of flexible Duran annuloplasty rings versus rigid Carpentier–Edwards rings, complete annuloplasty rings versus partial bands, small versus large anterior leaflet opening angle, and anterior leaflet tip opening length. Intraoperative echocardiography can measure the anterior leaflet opening angle, the anterior leaflet tip opening dimension, the MVA and the mean TMPG, and may help identify patients at risk for developing MS after MV repair.

## Introduction

The mitral valve (MV) complex plays a crucial role in cardiac function, controlling blood flow between the left atrium and the left ventricle. The components of the MV complex include an anterior leaflet, a posterior leaflet, tendinous chords, papillary muscles, and a saddle-shaped annulus at the atrioventricular junction (1). Deviation in the morphology of any of these components may result in compromised mechanical integrity of the MV complex, and abnormal leaflet coaptation (1). With studies suggesting that the MV is an active, dynamic structure that is susceptible to treatment, it is imperative to understand the types of diseases associated with these functional changes (2).

Mitral stenosis (MS) following MV repair surgery for non-rheumatic regurgitant MV disease is a pathology that is poorly understood. Currently, functional MS is defined as a mean transmitral pressure gradient (TMPG) >5 mmHg or a mitral valve area (MVA) <1.5 cm^2^ irrespective of the etiology (3, 4). Patients fall into two main categories; MS following ischemic mitral regurgitation (MR) repair or MS after degenerative MR repair. The occurrence of MS after MV repair is not well studied despite its relatively high incidence ranging from 9 to 54% (4–6). Furthermore, the impact of perioperative echocardiography on this condition is not clear (7). The 2014 ACC/AHA guidelines state that echocardiography should be used intraoperatively to guide MV repair and to ensure MV patency and correction of MR. However, the guidelines are based on studies of patients in the outpatient setting and make no comment of the potential detrimental effect of post-cardiopulmonary bypass (CPB) hemodynamic derangements on echocardiographic parameters (8). Consequently, patients may return a few years later after having improved in functional status following MV repair with newly developed MS (9). Therefore, it is vital to identify intraoperative echocardiographic parameters that may predict whether or not a patient will develop MS after MV repair surgery.

A few studies have investigated the development of MS after MV repair with conflicting results. Furthermore, a majority of the studies focused on the postoperative diagnosis of this condition without any mention of potential intraoperative contributing factors (10, 11). However, the identification of possible intraoperative predictors may be vital to patient outcomes. This article aims to provide an overview of the occurrence of MS after open MV repair surgery for regurgitant valves, its epidemiology, and risk factors. Likewise, we emphasize the role of the anesthesiologist in identifying intraoperative echocardiographic indices that may predict future development of MS. Our review excludes patients with rheumatic heart disease, patients with MS as the indication for MV surgery, and patients undergoing percutaneous MV repair.

## Etiology

Different mechanisms may lead to the occurrence of early or delayed MS after MV repair. These variables can be related to the surgical technique utilized for the repair (5). Early functional MS after MV repair was thought to be a direct result of the restrictive annuloplasty, since the elevated TMPGs are noticed early after the repair (4, 9). Repair surgery for degenerative MR involves resection of parts of the posterior leaflet, plication, and sliding. An annuloplasty ring or band is then placed to support the repair. Resection of the redundant leaflet tissue and the undersized rings may reduce the MV area and restrict its opening, resulting in obstruction at the levels of the annulus and leaflets (9).

Subvalvular tethering was suggested as a possible mechanism of functional MS following ring annuloplasty for ischemic MR in particular. This tethering restricts leaflet opening, and the annuloplasty ring reduces annular size and, consequently, further restricts MV opening, resulting in significant functional MS (12).

In cases of late fibrous tissue growth on the annuloplasty rings, pannus formation, as a possible foreign body reaction, was suggested as the cause of MS. Histopathological examination in such cases revealed giant cells containing intracytoplasmic ring material (13).

## Risk Factors

In 2002, Ibrahim and David published the first report in 4 out of 518 patients (14). The initial pathology for which the patients underwent MV repair was ischemic MR in two cases and degenerative MR in the other two cases. One interesting finding was that all four patients received a Duran ring annuloplasty for the initial repair. The MVA decreased in all patients from the preoperative value by 52–82%. Due to the symptomatic severe stenosis, the MV had to be replaced in three of the four patients, while the last patient had mild symptoms despite the low MVA (1.2 cm^2^) and needed no further intervention. Upon reoperation, extensive fibrous tissue was discovered on the annuloplasty ring. The pannus extended to the MV leaflets that became stiff, resulting in a narrowed opening (14).

In the years that followed, several reports were published on late MS due to pannus formation after MV regurgitation repair (13, 15–20). In most of these reports, MS developed after using a flexible Duran annuloplasty ring, with only one case developing with a rigid ring (17). In most cases, further echocardiographic assessment revealed that the MV leaflets were morphologically normal and the fibrous tissue predominantly formed at the level of the annuloplasty ring (18). In Nishida’s report, the fibrous tissue covered the Duran ring and also extended to the leaflets, which became stiff and immobile (16). Although this condition was often reported to develop in Duran ring recipients, the sporadic occurrence of such cases made the evaluation of risk factors very difficult (13, 15–20).

A recently published retrospective study compared a group of patients, mostly with degenerative MR, who received a Duran ring annuloplasty to another group that received a Carpentier–Edwards (CE) ring annuloplasty (21). The researchers found that the patients with the flexible Duran ring had significantly smaller MVA and higher mean TMPGs, resulting in a higher incidence of functional MS. Interestingly, Duran ring recipients experienced a gradual annual increase in mean TMPG at a rate of 0.19 mmHg/year, which was not experienced by the patients with the rigid CE ring. Significant pannus formation around the MV annulus could be detected on cardiac computed tomography (CT) and was significantly higher in the Duran ring patients, whereas the leaflet thickness was not different between the two groups, confirming that the obstruction was at the level of the annulus, and possibly caused by the pannus formation. These findings may indicate that MS after MV repair is a progressive process that starts early after the repair (21).

One explanation for the higher incidence of functional MS in Duran ring recipients is that Duran rings tend to be smaller than other ring types. Furthermore, the selection of Duran ring size is based on intertrigonal dimension, whereas for Carpentier rings, the selection is based on the intercommisural dimension, which is larger than the intertrigonal dimension (15, 20). Duran rings also have a circular non-physiological shape, which can alter the normal saddle shape of the mitral annulus (22). Additionally, the templates used to deliver Duran rings require excessive contact around the valve and may result in injury to the leaflets (15, 18).

Effective orifice area indexed to body surface area (EOAi) <0.9 cm^2^/m^2^ defines severe prosthesis-patient mismatch (PPM) after MV replacement, where the effective orifice area of the prosthetic valve is too small for the patient (23). Functional MS after MV repair may mimic PPM after MV replacement (3, 4). In a recent publication, Bertrand et al. observed an increase in EOAi during exercise in patients who underwent restrictive annuloplasty. An EOAi of >0.9cm^2^/m^2^, with exercise and not at rest, proved to be an independent predictor of functional capacity and outcome. The anterior leaflet opening angle (α) at peak exercise was the strongest determinant of EOAi (3).

In 2010, Kubota et al. (12) found that the anterior MV leaflet opening angle was an independent contributor to the leaflet tip opening length, which in itself, was an independent contributor to the MVA after repair. Although they did not offer any recommendations, the results showed a decrement in the opening angle from 68 ± 7 preoperatively to 62 ± 9 postoperatively and a decrease in the anterior leaflet tip opening dimension from 1.5 ± 0.2 cm preoperatively to 1.0 ± 0.3 cm postoperatively (*p* < 0.05). This was associated with an incidence of functional MS of approximately 42%. However, the findings in this study during exercise were against those of Bertrand’s. Although the annular size did not change, the mean and the peak TMPGs were significantly increased by exercise, whereas the MVA was significantly reduced. This reduction was attributed to further restriction of the anterior leaflet opening angle and the leaflet tip opening dimension (12). The role of post-CPB intraoperative EOAi and α-angle is still to be determined.

## Intraoperative Diagnostics

In the light of the aforementioned findings, it is crucial to identify the possibility of functional MS intraoperatively. Intraoperative echocardiographic assessment should ensure patency and rule out any restriction to the diastolic movement of the MV leaflets or any atrial flow convergence zone during diastole (24). The 2014 ACC/AHA guidelines recommend the use of 2D-planimetry, TMPG and pressure half-time (PHT) for the assessment of native MV area to establish the diagnosis, determine the severity, and guide the treatment of MS (8). However, there is a lot of controversy surrounding post-repair assessment of the MVA. The guidelines do not specify how to assess the MV during or after repair surgery to prevent a newly established MS.

The non-planar saddle shape of the mitral annulus plays an important role in the normal functioning of the MV. Mitral valve repair and placement of an annuloplasty ring may cause morphological changes to the annulus. Flat annuloplasty rings were found to increase the non-planarity angle (NPA), and thus making the annulus less non-planar, whereas the saddle-shaped rings were found to decrease the NPA and increase the non-planarity of the annulus (25). The innate non-planarity of the valve makes it hard to delineate the entire margin of the valve leaflets, and the morphological change after repair surgery further limits the ability of 2D-planimetry to assess the MVA (26, 27).

Nonetheless, in Maslow et al.’s report, MVA quantification by 2D-planimetry (1.0 cm^2^) and PHT (1.29 cm^2^) was consistent with MS, whereas peak TMPG was low (6 mmHg). Direct measurement of the gradient by inserting a needle into the left atrium and the left ventricle confirmed the presence of a high TMPG gradient of 16 mmHg, which prompted immediate revision of the valve (27).

On the other hand, Riegel et al. proposed that an intraoperative, post-CPB peak TMPG >17 mmHg or a mean TMPG >7 mmHg were suggestive of a clinically relevant iatrogenic MS that requires reoperation following MV repair. In their study, intraoperative MVA by PHT could not reliably predict the need for reoperation (28). The limitation of the PHT use is supported by other studies showing that echocardiography using PHT immediately after repair underestimates the measurement of MVA, creating a challenge when taking decisions regarding low estimated MVA. PHT is related not only to the MVA but also to the left ventricular and left atrial compliance and the flow and pressure gradients across the MV. Therefore, a change in transmitral blood flow, pressures, or chamber compliances after surgery may contribute to the measured PHT value, and may overestimate a non-significant MS (29).

After demonstrating the importance of MVA follow-up when predicting functional capacity (9), Chan et al. published another research paper looking at intraoperative TMPGs and MVA (30). They found that intraoperative mean and peak TMPGs were lower, and the planimetered MVAs were higher than the follow-up resting values (*p* < 0.05). However, their results showed that only intraoperative mean TMPGs, and not intraoperative MVAs, could predict the postoperative TMPG values. Furthermore, they proposed a linear correlation between intraoperative mean TMPGs (*x*) and follow-up mean TMPGs (*y*) as follows: (*y* = 1.8 + 0.86*x*). Their findings suggested that post-repair functional MS was predictable by an intraoperative mean TMPG value of >4 mmHg (30). Nonetheless, some studies argue against using TMPGs after repair surgery to make a diagnosis, as they are dependent on several variables and showing poor correlation with MVA (27, 31).

## Postoperative Functional Implications

### MS after Ischemic Mitral Regurgitation Repair

Most patients will experience improvement of their symptoms after MR repair, and the presence of a mild MS might not be recognized by the patient initially (4, 9). Magne et al. studied patients undergoing MV repair and CABG for ischemic MR (10). There was a significant increase in the postoperative mean and peak TMPGs (*p* < 0.001) and systolic pulmonary artery pressure (PAP) values (*p* < 0.008) compared to the preoperative measurements (10). When the study patients were compared to a control group of coronary artery disease patients without significant preoperative MR, the mean TMPGs and PAP were found to be significantly higher in the annuloplasty group both at rest and during a dobutamine stress test (*p* < 0.05). The annuloplasty group also had significantly smaller MV effective orifice areas (EOA) postoperatively (*p* < 0.05). This group also showed a significant reduction in 6-minute walking test (6MWT) distance 13 ± 3 months postoperatively. Furthermore, 54% of the annuloplasty patients had a postoperative MVA <1.5 cm^2^ and a mean TMPG >5 mmHg. The PAP and exercise TMPG findings in the annuloplasty group were similar to those found in patients with moderate-to-severe MS. The authors concluded that the findings suggested functional MS development after MV annuloplasty, and that this MS resulted in reduced patient functional capacity (10).

Rubino et al. found different results when they compared patients with ischemic MR who had post-repair mean TMPGs >5 versus <5 mmHg 9 months postoperatively (11). Although approximately 50% of the patients showed at least mild MS post-surgery, the two groups were not significantly different in long-term freedom of hospitalization, freedom of congestive heart failure, and 23-month survival. Contrary to Magne’s paper, which found smaller MVA in the group with higher TMPGs, Rubino et al. found that there was no difference in MVA between the two groups. The authors concluded that functional MS induced by restrictive annuloplasty was well tolerated by the patients and did not impact patient survival or functional capacity (11).

These findings were further supported by another study, showing that after MV repair for ischemic MR, mean TMPGs >5 mmHg did not adversely affect patient survival (5). On the contrary, it was discovered that mean TMPGs >5 mmHg were associated with a higher maximum oxygen uptake, a higher cardiac output (CO), and an increase in exercise capacity. They proposed that TMPGs in patients with previous ischemic MR should be read in light of CO and LV function values; a high TMPG in the presence of a normal CO might be acceptable, whereas low TMPGs may mask a significant MS in a low CO patient (5).

### MS after Degenerative Mitral Regurgitation Repair

Chan et al. compared patients undergoing annuloplasty for degenerative MR, with high postoperative resting TMPGs to a control group with low resting TMPGs (9). They found that the group with the higher gradients also had significantly higher left atrial volumes at rest (*p* = 0.02) and smaller calculated MVA on echocardiography. The PAP also was higher both at rest and peak exercise in the high gradient group (*p* = 0.02) and correlated with the resting mean TMPG. Their findings were consistent with a functional MS and multivariable analysis showed that MVA was an independent predictor of exercise capacity (9).

Mesana et al. found similar results when they compared patients who received partial band annuloplasty to patients who received a complete ring annuloplasty (32). In that study, patients who underwent annuloplasty using the complete ring had higher postoperative mean TMPGs both at rest and exercise (*p* < 0.0001 and *p* = 0.0002, respectively). They also had higher right ventricular systolic pressures both at rest (*p* = 0.003) and peak exercise (*p* = 0.004), greater indexed left atrial volumes (*p* < 0.001), and smaller MVA (*p* < 0.001). The difference in mean TMPGs between the two groups was even more prominent at peak exercise. On functional assessments, patients with a complete ring annuloplasty reported lower overall general health (*p* = 0.007) and energy levels compared to partial band patients (*p* = 0.02). This study faces a noteworthy limitation that requires further investigation. The complete ring group was a mix of patients who were randomly assigned to receive either a Duran ring or a CE ring. The difference between the two groups might be attributable to the higher risk of MS associated with Duran rings, and not the fact that the rings were complete or partial (32).

## Conclusions

Mitral stenosis after MR repair surgery is a problem that originates from the time of surgery and progresses over time. It is usually not detected before the patient leaves the operating room due to the lack of adequate studies and guidelines that can help diagnose this problem intraoperatively. Most patients with severe MR who live sedentary lives will improve after the repair and tolerate the increased TMPGs and narrowed MVA without developing symptoms for many years. Young individuals often undergo MV repair to benefit from the functional improvement that surgery offers and avoid future complications. Therefore, MV repair surgery should never trade regurgitation for a new stenosis. The collaboration between the cardiac surgeon performing the procedure and the anesthesiologist monitoring the intraoperative variables is paramount. Based on the current literature, no strong recommendations can be drawn regarding early prediction. Future studies should recognize and validate the red flags that indicate which individuals are at a higher risk for developing MS. Intraoperative post-CPB echocardiographic assessment of the anterior leaflet opening angle and opening dimension, accurate measurement of MVA, and possible changes to the surgical techniques may prove beneficial. Cardiac anesthesiologists are encouraged to consider establishing specific guidelines that can help prevent MS after repair surgery for MR patients.

## Author Contributions

MS, CL, BB, MD, and SK conducted a review of the literature. MS, CL, BB, SK, and NK prepared the body of the manuscript. ME critically reviewed the publication. All authors endorsed the final form of the manuscript.

## Conflict of Interest Statement

The authors declare that the research was conducted in the absence of any commercial or financial relationships that could be construed as a potential conflict of interest.
